# Sex and self defense

**DOI:** 10.7554/eLife.73723

**Published:** 2021-10-14

**Authors:** Milton T Drott

**Affiliations:** Department of Medical Microbiology and Immunology, University of Wisconsin-Madison Wisconsin-Madison United States

**Keywords:** *Aspergillus nidulans*, verticillium, sordaria macrospora, folsomia candida, trichorhina tomentosa, fungi, Other

## Abstract

The fungus *Aspergillus nidulans* produces secondary metabolites during sexual development to protect itself from predators.

**Related research article** Liu L, Sasse C, Dirnberger B, Valerius O, Fekete-Szücs E, Harting R, Nordzieke DE, Pöggeler S, Karlovsky P, Gerke J, Braus GH. 2021. Secondary metabolites of Hülle cells mediate protection of fungal reproductive and overwintering structures against fungivorous animals. *eLife* **10**:e68058. doi: 10.7554/eLife.68058

When walking through the woods we often look up, focusing on the birds and the rustling leaves in the canopy above us, but on the ground a drama is unfolding: the fungi are under attack. Looking down you may see a slug grazing on mold that has established itself on an old log, or a mushroom swarmed by fruit flies on the forest floor. So how do fungi protect themselves from these attacks if they cannot physically escape?

Previous research has shown that fungi defend themselves using secondary metabolites, chemical compounds which are not essential for growth but involved in ecological interactions (Janzen, 1977; Rohlfs and Churchill, 2011). These compounds can be toxic to animals and/or drive them away from the fungus. As predators can appear without warning, fungi must be ready with the metabolites at short notice, either by making them ahead of time, or by rapidly creating them in response to a threat (Drott et al., 2017). However, even in the best-studied fungi, it is unclear exactly where and when these defensive chemicals are created, making it difficult to fully understand their ecological purpose.

Now, in eLife, researchers from the University of Göttingen – including Li Liu and corresponding authors Jennifer Gerke and Gerhard H Braus – report that a set of previously identified secondary metabolites called xanthones (Sanchez et al., 2011) are produced during certain life stages of the soil-dwelling fungus *Aspergillus nidulans* (Liu et al., 2021). Xanthones are synthesized through a series of chemical reactions controlled by a group of genes known as the *mdp/xpt* cluster. The proteins encoded by the *mdp* genes make the ‘backbone’ of the metabolite, which is then progressively modified by proteins produced from the *xpt* genes until the final compound is formed.

To narrow down where xanthones are synthesized in the fungus, Liu et al. added a fluorescent tag to the protein responsible for the final chemical reaction, as this represents the complete synthesis of the secondary metabolites produced by the *mdp/xpt* pathway. This revealed that xanthones are created in Hülle cells which support the development of cleistothecia, fruiting bodies that allow the fungus to sexually reproduce and last through the winter (Troppens et al., 2020). This suggests that xanthones are not produced throughout the life of the fungus, but are only generated during the stages of the fungus’ sexual lifecycle when cleistothecia form.

Next, Liu et al. set out to determine the role of other genes in the *mdp/xpt* cluster by creating a set of mutant fungi that are missing one of these genes. They found that each gene plays a specific role in the sequence of chemical reactions that synthesize the xanthones used by the fungi. As a result, none of the mutant strains were able to produce the final xanthones, and instead accumulated intermediate chemical structures that are generated during this pathway. Like xanthones, these intermediates only appeared at times when the fungus was forming cleistothecia.

It is clear from these findings that *A. nidulans* likely uses xanthones during sexual development; but what role do these secondary metabolites play in ecology? To investigate this, Liu et al. grew fungal colonies and cleistothecia from mutated and non-mutated (or wild-type) strains of *A. nidulans* and exposed them to arthropods (invertebrates with exoskeletons such as insects and arachnids) that eat fungi (Figure 1). Wild-type colonies – which can produce all of the xanthones – were damaged less heavily by the arthropods than the mutant colonies. Further experiments showed that, in addition to mitigating damage from arthropods, some of the intermediates formed during *﻿*synthesis can suppress fungal growth when added to other fungi in the laboratory. However, these intermediates did not accumulate to high levels in the wild-type strain and also suppressed the development of *A. nidulans*, raising doubts about their potential benefit to the fungus when competing with other fungi in nature.

**Figure 1.**
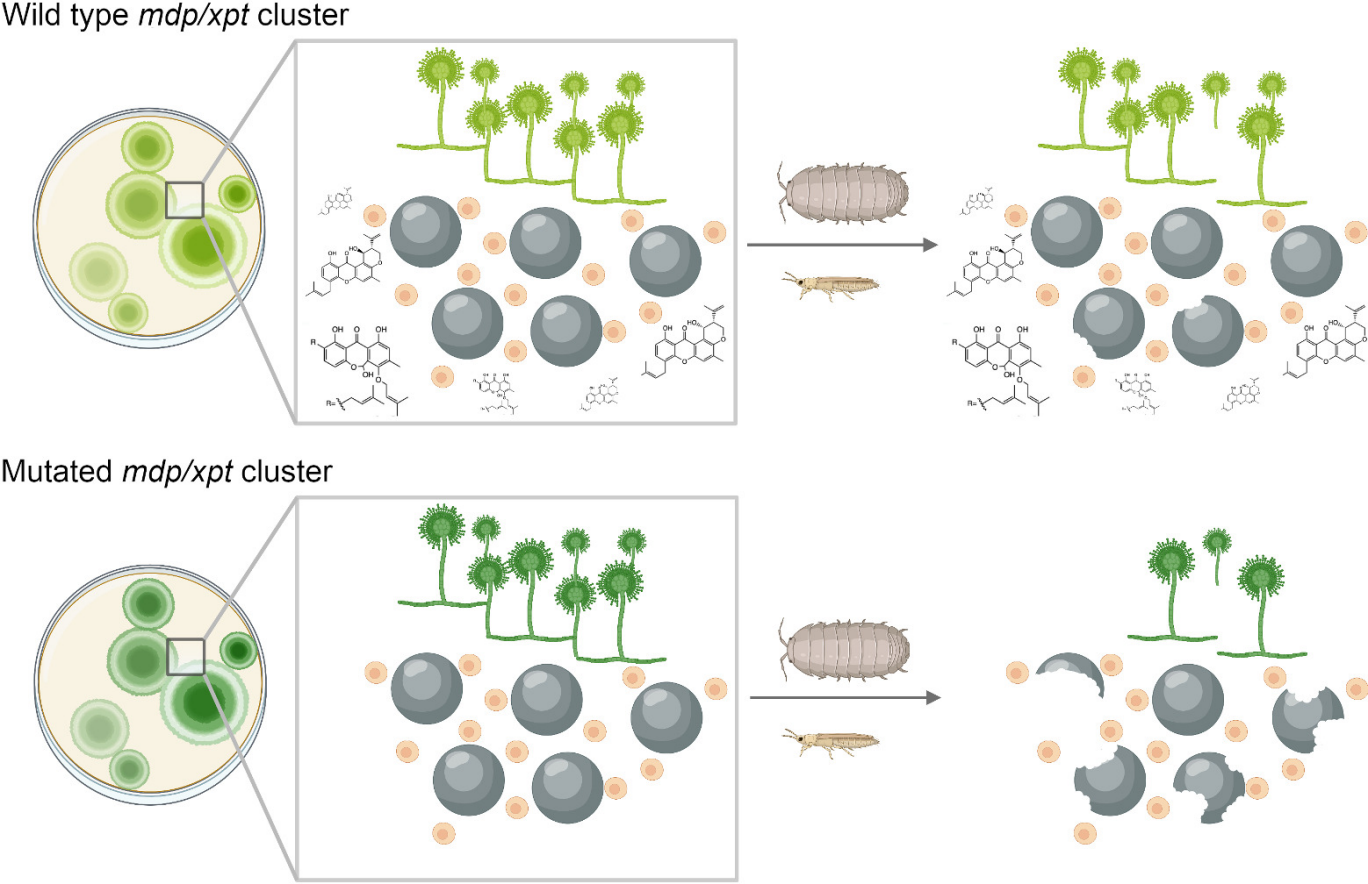
Chemical products of the *mdp/xpt* gene cluster protect *A. nidulans* from predators. *A. nidulans* produces secondary metabolites called xanthones using a set of genes known as the *mdp/xpt* cluster. (Top) In the wild-type fungus, xanthones (shown as chemical structures) are produced by Hülle cells (small beige circles) and then accumulate in sexual fruiting bodies called cleistothecia (large black circles). When arthropods attack the wild-type fungus, the xanthones deter these predators and stop them from destroying the cleistothecia. (Bottom) Fungi with lab-induced mutations in the *mdp/xpt* genes are unable to produce xanthones, making them more susceptible to fungus-eating arthropods.

Image credit: Milton T Drott; Figure created using BioRender.com.

Hülle cells are found in other fungi (Dyer and O'Gorman, 2012), and genes resembling the *mdp/xpt* cluster occur in other species where no sexual cycle has been observed to date (de Vries et al., 2017). It remains to be seen how secondary metabolites that appear at specific life stages – like the ones in this study – translate into these other species. Furthermore, it is unclear how these chemical compounds relate to previous observations that other secondary metabolites with unknown functions are only produced under certain conditions (Georgianna et al., 2010). The findings of Liu et al. emphasize the complicated interplay between fungi and their environment, and spark further questions about about how the fungus' investment in protecting its sexual offspring has impacted its evolution.
